# Transfer of metals from soil to vegetables and possible health risk assessment

**DOI:** 10.1186/2193-1801-2-385

**Published:** 2013-08-15

**Authors:** Yeasmin Nahar Jolly, Ashraful Islam, Shawkat Akbar

**Affiliations:** Chemistry Division, Atomic Energy Centre, P.O. Box 164, Dhaka, 1000 Bangladesh; Nuclear Power and Energy Division, Atomic Energy Commission, Dhaka, 1207 Bangladesh

**Keywords:** Daily intake, Hazard quotient, Health risks, Metal contamination, Transfer factors

## Abstract

Metal contamination in agricultural soils is of increasing concern due to food safety issues and potential health risks. Accumulation of Heavy and trace metals in vegetables occur by various sources but soil is considered the major one. Consumption of vegetables containing (heavy/trace) metals is one of the main ways in which these elements enter the human body. Once entered, heavy metals are deposited in bone and fat tissues, overlapping noble minerals and cause an array of diseases. The present study aimed to investigate the concentration of different metals in agricultural soil and vegetables grown on those soils and to evaluate the possible health risks to human body through food chain transfer. Contamination levels in soils and vegetables with metals were measured and transfer factors (TF) from soil to vegetables and its health risk were calculated accordingly. Results showed that concentration of Si, Ba, K, Ca, Mg Fe, Sc, V, Cr, Cu, Zn, As, Mn, Co, Ni, Se, Sr, Mo, and Cd in soil is higher than the World Average value and Al, Ti and Pb is lower than the World Average value whereas concentration of toxic elements like As, Co, Cu, Mn, Pb, Se, Ni, V and Zn in vegetable samples are below the World Average value. The intake of toxic metals (Fe, Cu, Mn, Zn Co, Cr, V, Ni, Pb and Cd) from vegetables is not high and within the permissible limit recommended by WHO, Food & Nutritional Board and US EPA. The Hazard Quotient (HQ) for Fe, Cu, Co, Cr, V, Ni, Pb, Mn, Zn and Cd were calculated which showed a decreasing order of Cd>Mn>Zn>Pb>Cu>Fe>Ni>V=Co>Cr. Highest HQ value found for Cd (2.543) which is above the safe value.

**Electronic supplementary material:**

The online version of this article (doi:10.1186/2193-1801-2-385) contains supplementary material, which is available to authorized users.

## Introduction

The accumulation of heavy metals and metalloids in agricultural soil is of increasing concern now a day. Potentially harmful metal in soil may come from the bedrock itself and anthropogenic sources like solid or liquid waste deposits, agricultural inputs and fallout of industrial and urban emissions (Wilson and Pyatt 2007). Excessive accumulation in agricultural soils results in soil contamination and has consequences for food quality and safety.

Food is the major intake source of toxic metals by human beings. Among food system, vegetables are the most exposed food to environmental pollution due to aerial burden. Vegetables take up heavy metals and accumulate them in their edible and non-edible parts at quantities high enough to cause clinical problems to both animals and human beings. Excessive content of metals beyond Maximum Permissible level (MPL) leads to number of nervous, cardiovascular, renal, neurological impairment as well as bone diseases and several other health disorders (WHO 1992; Steenland and Boffetta 2000; Jarup 2003).

Vegetables are an essential part of diet and are taken both cooked and raw forms by human. Vegetables act as buffering agents for acid generation during digestion (Maleki and Zarasvand 2008) and some metals present in vegetables are even important biochemically and psychologically from health point of view. Metals like cobalt (Co), chromium (III) (Cr), copper (Cu), iron (Fe), manganese (Mn), molybdenum (Mo), selenium (Se) and zinc (Zn) help in regulating human metabolism (Lokeshappa et al. 2012). Manganese is an essential elements act as an activator and constituent of many enzymes present in human (Sresty and Rao 1999). But some elements like Pb, Cd, As are very toxic for human. Lacatusu et al. (Lacatusu et al. 1996) reported that soil and vegetables contaminated with Pb and Cd in Copsa mica and Baia Mare, Romania, significantly contributed to decrease human life expectancy (9–10 years) within the affected areas. Other elements such as Cr, Co, and Ni although essential for human but at concentrations higher than those recommended may cause metabolic disorders.

Soil to plant transfer of heavy metals is the major path way of human exposure to metal contamination. The present study conducted measurement of heavy and trace metal level in the soil collected from Rooppur, Pabna as well as vegetables grown on the soil and determination of transfer factor of these elements to evaluate potential health effect of the people those who consumes those vegetables.

## Materials and methods

### Sample collection and preparation

**Figure 1:**
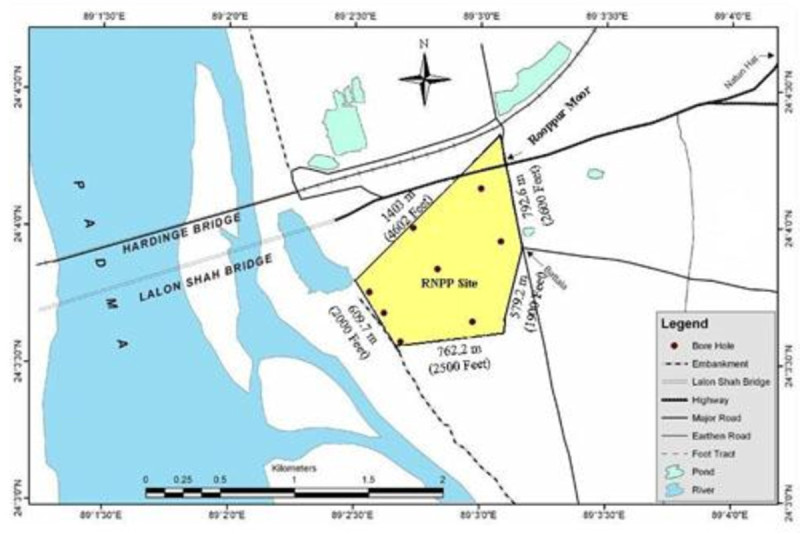
**Map of the Sample Location.**

Ten Soil samples were collected from the agricultural land at Ruppur area of Pabna District of Bangladesh and leveled as SS (SS1-SS10). The geographical coordinates of Ruppur area is 24° 4’ 0” North, 89° 2’ 0” East. It is situated by the side of river Padma. Available vegetables grown on those sites were collected as well. The detailed of the vegetable samples collected from the sites are given in Table 1 and the map of the sampling site is shown in Figure 1. The collected soil samples were dried at 60°C in an oven until constant weight was obtained. The dried samples were ground to fine power in an Agate mortar with a pestle and preserved in polyethylene bags in a desiccator for further analysis. The vegetable samples were first thoroughly washed with tap water and finally with deionized water. The samples were then dried in an oven at 60°C until constant weight was obtained. The dried samples were finally ground in a carbide mortar with a pestle and preserved in polyethylene bags in a desiccator until subsequent analysis.

**Table 1:** **Description of vegetable samples analyzed**

Common name	Designation	Scientific name	Edible part
Spinach	SP	*Beta vulgaris* L	Leaf
Amaranthus	AM	*Amaranthus caudatus* L	Leaf
Brinjal	BR	*Solanum melongena* L	Fruit
Tomato	TO	*Lycopersicon esculentum* L	Fruit
Radish	RA	*Raphanus sativus* L	Root
Bean	BN	*Phaseolus lunatus* L	Fruit
Cauliflower	CF	*Brassica oleracea* L	Inflorescence
Carrot	CA	*Daucus carota var sativus* L	Root

### Methods of analysis

The Panalytical Epsilon 5 Energy Dispersive X-ray Fluorescence (EDXRF) was used as major analytical technique for carrying out elemental analysis in the samples. The Panalytical Epsilon 5 EDXRF Spectrometer System is a fully integrated X-ray analysis system, combining a unique energy dispersive X-ray fluorescence spectrometer, with instrument control, analysis software and programmable automatic sample changer system was used. The spectrometer is provided with a low energy Gd X-ray tube of max. power 600 W to generate X-ray beam and a PAN-32 30 mm^2^ Ge X-ray detector with the energy resolution of <140 eV at 5.9 keV for detection of elemental characteristic X-rays emitted from the excited materials.

### Sample irradiation with X-ray beam

For irradiation of the sample with X-ray beam 2 g of each powdered material was pressed into a pellet of 25 mm diameter with a pellet maker (Specac) and loaded into the X-ray excitation chamber with the help of automatic sample changer system. The irradiation of all real samples were performed by assigning a time-based programme, controlled by a software package provided with the system. The standard materials were also irradiated under similar experimental conditions for construction of the calibration curves for quantitative elemental determination in the respective samples. The generated X-ray spectra of the materials were stored into the computer.

#### Calculation of X-ray intensities

The X-ray intensities of the elements in sample spectrum were calculated using the system software by integration of area of the respective X-ray peak areas using peak fitting deconvolution software.

### Data analysis

#### Calculation of oral intake of metals from soil through vegetables

Calculation of oral intake of metals from soil through vegetables was calculated following Cui et al. (2004).

Daily intake of metals (DIM) = daily vegetable consumption×mean vegetable metal concentrations (mg/day, fresh weight).

The required amount of vegetables in our daily diet must be 300 to 350 g per person has been suggested by WHO guideline (WHO 1998).

#### Calculation of health risk index of metal contamination of vegetable

Risk to human health by the intake of metal-contaminated vegetables was characterized using a hazard Quotient (HQ) (U.S. Environmental Protection Agency US EPA 1989). HQ is the ratio between exposure and the reference oral dose (R_f_D). If the ratio is lower than one (1), there will be no obvious risk. An estimate of the potential hazard of metal to human health (HQ) through consumption of vegetables is determined by the following equation

$$\mathrm{HQ}=\left(\mathrm{Div}\right)\times \left(C_{\mathrm{metal}}\right)/R_{f}D\times \mathrm{Bo}$$

Where (Div) is the daily intake of vegetables (kg/day), (C_metal_) is the concentration of metal in the vegetable (mg/kg), R_f_D is the oral reference dose for the metal (mg/kg of body weight/day) and Bo is the human body weight (kg). Although the HQ-based risk assessment method does not provide a quantitative estimate for the probability of an exposed population experiencing a reverse health effect, it indeed provides an indication of health risk level due to exposure to pollutants (Chary et al. 2008).

#### Transfer factor

Metal concentration in the extracts of soils and plants were calculated on the basis of dry weight. The plant transfer factor (TF) was calculated as follows:

$$\begin{array}{l} \;\mathrm{TF}=C_{\mathrm{plant}}/C_{\mathrm{Soil}} \end{array}$$

Where *C*_*plant*_ and *C*_*soil*_ represents the toxic metal concentration in extracts of plants and soils on dry weight basis, respectively.

## Results and discussion

### Concentration calibration

A direct comparison method based on EDXRF technique was used for elemental concentration measurement (Islam and Jolly 2007; Jolly et al. 2012). As the analysis is based on direct comparison, the standards of similar matrices were used for the construction of the calibration curve in order to avoid the matrix effect. Three soil standards (Soil-7/IAEA, Montana-1/2710a, Montana-2/2711a) and five plant standards (Apple Leaf/NIST 1516, Spinach/NIST 1570a, Orchard Leaf/NIST 1571, Tomato Leaf/NIST 1573a, and Peach Leaf/NIST 1574) were used for the construction of calibration curves for carrying out elemental analysis in soil and plant respectively. The calibration curve for each element was constructed based on the K X-ray or L X-ray intensities calculated for the respective elements present in standard samples. The curves were constructed by plotting the sensitivities of the elements as a function of their atomic number.

The validation of calibration curves constructed for elements present in the standards was checked through analysis of standard reference materials. The results obtained for elements of interest and certified values for corresponding elements are shown in the Table 2. All results in respect of certified known values were found to vary within the acceptable range of error.

**Table 2:** **Comparison between present results and the certified values of standard reference materials (mg kg** ^**-1**^ **)**

Elements	Soil (Montana- 1)			Plant (spinach)		
	Results obtained	Certified values	Error	Results obtained	Certified values	Error
K	21113	21700	2.71	27729	29030	2.26
Ca	9136	9640	5.23	14483	15270	4.92
Mn	2128	2140	0.56	69.33	75.90	0.76
Fe	39685	43200	8.14	-	-	-
Ni	8.67	8.0	−8.38	-	-	-
Cu	3409	3420	0.32	13.30	12.20	−8.98
Zn	4179	4180	0.02	-	-	-
As	1441	1540	6.43	0.035	0.038	9.21
Se	1.2	1.0	−20.0	0.053	0.050	6.00
Pb	5382	5520	2.5	-	-	-

### Concentration of major and toxic element in soil

**Figure 2:**
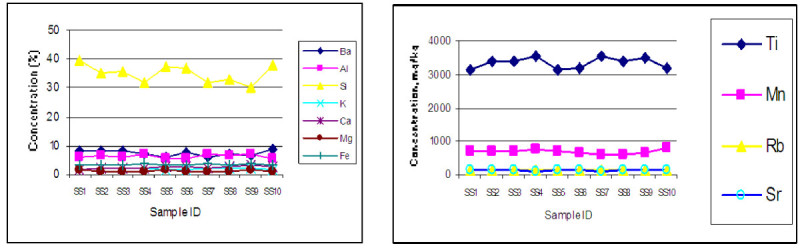
**Variation of concentration of major elements (Ba, Al, Si, K, Ca, Mg, Fe) and (Ti, Mn, Rb, Sr) in soil with location.**

**Figure 3:**
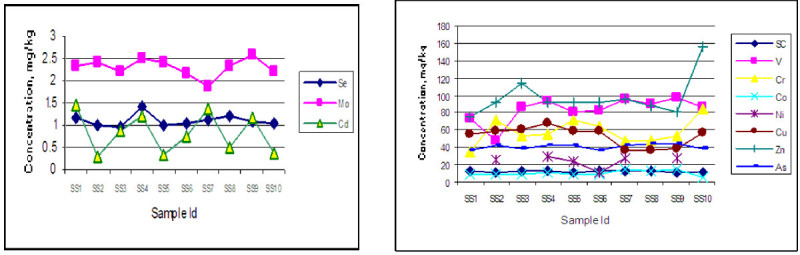
**Variation of concentration of toxic elements (Se, Mo, Cd) and (Sc, V, Cr, Ni, Cu, Zn, As) in soil with location.**

The average concentration of major elements Al, Si, Ba, K, Ca, Mg, Ti and Fe of the surface soil are found 6.22, 35.06, 7.40, 2.10, 2.51, 1.25, 0.33, 3.43% respectively whereas the average concentration of toxic elements like Sc, V, Cr, Cu, Zn, As, Mn, Co, Ni, Se, Sr, Mo, Pb and Cd are found 12.01, 8.4, 58, 53, 98, 41, 691, 10.18, 23.81, 1.11, 142, 2.30, 15.0 and 0.84 mg kg^-1^ respectively. According to (Pendias and Pendias 2000) concentration of most of the elements except Al, Ti, Pb in the measured soil is higher than the World Average value. The elemental concentration in the soil follows a similar trend but varied according to the sampling location as shown in Figures 2 and 3

### Concentration of major and toxic element in vegetable samples

The mean concentration of macro & micronutrients and toxic elements showed variations among different vegetables collected from the sites (Table 3). Table 4 showed the relative abundance of different elements in the Agricultural product. Macronutrients (P, K, Ca and Fe) are abundant in all type of vegetables. Calcium is present in higher concentration in leafy vegetables than others. Iron is present in small quantities in all types of samples compared to other macronutrient and lowest concentration is found in Brinjal. Lokeshappa et al. (2012) studied the elemental concentration in different agricultural product and the results obtained are comparable with the present study. Among the micronutrient Cu and Zn is abundant in all varieties and concentration of zinc is high comparable to copper. Mn is present in Bean only with moderate concentration (26 mg/kg). Vanadium is present in spinach, amaranthus, carrot and radish while Cr is present in radish, amaranthus tomato and cauliflower in lower concentration. Co and Ni are found only in the leafy vegetables with lower concentration and within the permissible limit (Codex General Standard for Contaminants and Toxins in Food and Feed 1995).Toxic element like As (0.08-0.04 mg/kg), Se (0.20-0.03 mg/kg) and Pb (0.98-0.13 mg/kg) is present in almost all varieties of samples but concentration of As and Se is very low to contribute any toxic effect. On the other hand Pb showed comparatively higher value than the others which may be attributed to plants grown on the agricultural lands located near high ways. Sr and Cd are present in the leafy vegetable (amaranthus and spinach) in moderate concentration and ranges from 7.23 to 61.65 and 0.65 to 0.97 mg kg^-1^ respectively. Hence Cadmium is a dangerous element because it can be absorbed via alimentary tract; penetrate through placenta during pregnancy and damage membranes and DNA. (Wagner 1993) reported that vegetables may contribute to about 70% of Cd intake by human varying according to the level of consumption.

**Table 3:** **Concentration of different elements in vegetable samples**

Elements	Concentration, mg/kg							
	Spinach	Amaranthus	Brinjal	Tomato	Radish	Bean	Cauliflower	Carrot
P	2829 ±302	6306 ±259	5293 ±304	4716 ±323	6867 ±343	76444±335	6976 ±333	5108 ±317
K	32750 ±421	3506 ±399	23623 ±447	27924±467	29975 ±486	23273±453	30308 ±442	28581±425
Ca	12717 ±334	21858 ±353	4724 ±372	5050 ±334	7443 ±331	10039±345	6581 ±356	6441 ±342
V	0.26 ±0.08	0.15 ±0.09	<0.04	<0.04	0.11 ±0.07	<0.04	<0.04	0.13 ±0.07
Cr	<0.05	1.15 ±0.04	<0.05	0.51±0.03	1.68 ±0.08	<0.05	0.47 ±0.02	<0.05
Mn	<0.06	<0.06	<0.06	<0.06	<0.06	25.95 ±2.56	<0.06	<0.06
Fe	210.30 ±1.28	195.00 ±1.02	32.60 ±0.99	43.86 ±0.84	40.89 ±0.79	97.69 ±0.85	60.01 ±0.74	58.77 ±0.77
Co	0.35 ±0.09	0.37 ±0.07	<0.27	<0.27	<0.27	<0.27	<0.27	<0.27
Ni	<0.65	<0.65	<0.65	<0.65	0.87 ±0.13	<0.65	0.94 ±0.29	<0.65
Cu	5.59 ±0.33	4.87 ±0.35	6.69 ±0.37	3.62 ±0.29	4.45 ±0.34	5.91 ±0.22	4.59 ±0.35	5.35 ±0.31
Zn	112.24 ±0.47	55.42 ±0.45	40.14 ±0.43	31.1 ±0.43	25.78 ±0.46	68.34 ±0.44	42.05 ±0.43	45.28 ±0.45
As	<0.01	0.08 ±0.01	0.05 ±0.00	0.05 ±0.00	0.05 ±0.00	0.05 ±0.00	0.05 ±0.00	0.04 ±0.00
Se	0.08 ±0.02	0.03 ±0.00	0.16 ±0.01	0.14 ±0.01	0.10 ±0.00	0.08 ±0.00	0.20 ±0.01	0.09 ±0.00
Sr	23.75 ±0.23	61.65 ±0.38	<0.14	<0.14	7.23 ±0.28	<0.14	<0.14	<0.14
Cd	<0.06	0.97 ±0.07	<0.06	<0.06	0.65 ±0.05	<0.06	0.16 ±0.04	<0.06
Pb	0.98 ±0.00	0.96 ±0.01	0.83 ±0.14	0.12 ±0.00	0.51 ±0.06	0.65 ±005	0.23 ±0.00	0.72 ±0.03

**Table 4:** Relative abundance of different elements in the vegetables

Relative abundance of macronutrient in the vegetables	
P	BN>CA>RA>AM>BR>CA>TO>SP
K	SP>CF>RA>CA>TO> BR>BN>AM
Ca	AM>SP>BN>RA>CF>CA>TO>BR
Fe	SP>AM>BN>CF>CA>TO>RA>BR
**Relative abundance of micronutrient in the vegetables**	
V	SP> AM> CA> RA> Remaining in BDL (<0.04 mg kg^-1^)
Cr	RA> AM> TO> CF> Remaining in BDL (<0.05 mg kg^-1^)
Mn	BN> Remaining in BDL (<0.06 mg kg^-1^)
Co	AM> SP> Remaining in BDL (<0.27 mg kg^-1^)
Ni	CF >RA> Remaining in BDL (<0.65 mg kg^-1^)
Cu	BR >BN >SP> CA> CF >AM>RA> TO
Zn	SP> BN >AM> CA> CF> BR> TO >RA
**Relative abundance of toxic element in the vegetables**	
As	AM> TO> RA =CF> BR> BN=CA>Remaining in BDL (<0.01 mg kg^-1^)
Se	CF >BR> TO> RA> CA> BN> SP> AM
Sr	AM> SP> RA> Remaining in BDL (<0.14 mg kg^-1^)
Cd	AM> RA> Remaining in BDL (<0.06 mg kg^-1^)
Pb	SP >AM> BR> CA> BN> RA> CF> TO

### Transfer factors (TF) from soil to vegetables

The TF values for K, Ca, V, Cr, Mn, Fe, Co, Ni, Cu, Zn, As, Se, Sr, Cd, and Pb for various vegetables varied greatly between plant species and locations (Table 5).From the Table 5 it is observed that K, Ca and Zn has higher transfer factor for all types of vegetables and ranges from 0.167 (*Amaranthus caudatus* L) to 1.559 (*Beta vulgaris* L), 0.189(*Solanum melongena* L) to 0.872 (*Amaranthus caudatus* L) and 0.263 (*Raphanus sativus* L) to 1.148 (*Beta vulgaris* L) respectively. Transfer factor for V, Cr, Mn, Fe, Co, Ni, and As is very low compared to other elements in all varieties of vegetables. The TF value for Se is quite high in most of the vegetables and ranges from 0.031 (*Amaranthus caudatus* L) to 0.147 (*Solanum melongena* L) and the TF value for Cu (0.069-0.127) and Sr (0.051-0.433) is medium. The TF value for toxic element Cd (0.192-1.161) is quite high compared to Pb (0.008-0.065) but Cd is found only in three varieties of vegetables. The leafy vegetables are found to show a higher transfer factor among the studied vegetables (Table 5). The present result agrees with the investigation made by (Zhuang et al. 2009) in the food crops in the vicinity of Dabaoshan mine, South China where the Bio-accumulation factors for heavy metals were significantly higher for leafy than non-leafy vegetables. Soil electrolyte plays an important role in the process of metal transfer. The electrochemical properties of soil reflected through the temperature, pH, and electrolyte concentration etc. thus influenced the migration transformation ability of toxic metal indirectly. In a study, (S. Satter 2012) found that the transfer of Zn and Pb from soil of Tegharia union at Keraniganj, Dhaka, Bangladesh to plant *Enhydra fluctuans* and *Oryza sativa* is 1.762 and 1.05; and 5.519 and 1.20 respectively which is quite high, similarly transfer factor (TF) of Mn is also high in *Enhydra fluctuans* (1.553). As these two plants are widely consumed by human, through these plants toxic elements can be transferred to human body creating disruption in various biological systems. Therefore, workers and the residents of theses areas are in high health risks of toxic metal exposure.

**Table 5:** **Transfer factor from soil to vegetables**

Elements	Transfer factor							
	Spinach	Amaranthus	Brinjal	Tomato	Radish	Bean	Cauliflower	Carrot
K	1.559	0.167	1.124	1.329	1.426	1.108	1.442	1.360
Ca	0.507	0.872	0.189	0.202	0.300	0.401	0.262	0.257
V	0.003	0.002	-	-	0.001	-	-	0.002
Cr	-	0.02	-	0.009	0.029	-	0.008	-
Mn	-	-	-	-	-	0.038	-	-
Fe	0.006	0.006	0.001	0.001	0.001	0.003	0.002	0.002
Co	0.035	0.036	-	0.023	-	0.02	0.025	0.020
Ni	-	-	-	-	0.037	-	0.039	-
Cu	0.106	0.092	0.127	0.069	0.084	0.112	0.087	0.101
Zn	1.148	0.567	0.411	0.318	0.263	0.699	0.430	0.463
As	-	0.002	0.001	0.001	0.001	0.001	0.001	0.001
Se	0.069	0.031	0.147	0.126	0.088	0.072	0.178	0.081
Sr	0.167	0.433	-	-	0.051	-	-	-
Cd	-	1.161	-	-	0.778	-	0.192	-
Pb	0.065	0.064	0.055	0.008	0.034	0.043	0.015	0.048

### Daily intake of metals by human beings from mixed vegetable

Table 6 shows approximate daily intake of metals by human beings from mixed vegetables. The intake values are calculated by taking the average value of metals in all the eight varieties of the vegetables (Table 1) and considering that each person (assuming 70 kg of body weight) consumes approximately 300 g (WHO 1998) of vegetables per day. Hence different vegetables are consumed variably by different segment of population at different time throughout the year, so it may be a realistic estimate for the average intake of metals from vegetables. It may be how ever be seen in Table 6 that intake of toxic metals except Mn, Zn and Cd from vegetables is not high and within the permissible limits recommended by various agencies (Friberg et al. 1984; Food and Nutritional board 2004; U.S. Environmental Protection Agency [US EPA] 2010; World Health Organisation [WHO] 1993; World Health Organisation [WHO] and WHO 2004). However intake of Mn and Zn is comparable to the suggestive value but value of Cd is really alarming.

**Table 6:** **Estimated Daily Intake of Metal (DIM) through vegetables**

Trace elements	Average conc. Of 8 vegetables (μg/g)	Intake by human being (mg/g)	R_f_D^a^(mg/day)	References
Fe	92.390	27.72	10.0-60.0	WHO 1994
Cu	5.134	1.54	2.0-3.0	WHO 1994
Mn	25.950	7.79	0.5-5.0	WHO 1994
Zn	52.544	15.76	15.00	WHO 1994
Co	0.268	0.080	3.010	Food and Nutrition Board, 2004
Cr	0.953	0.286	105	US EPA 2010
V	0.161	0.048	1.80	WHO 2004
Ni	0.905	0.272	1.400	US EPA 2010
Pb	0.625	0.188	0.245	WHO 1993
Cd	0.593	0.178	0.070	US EPA 2010

^a^R_f_D is the oral reference dose for the metal (mg/kg body weight/day).

### Potential hazard of metal to human health (HQ)

The Hazard Quotient (HQ) for Fe, Cu, Co, Cr, V, Ni, Pb, Mn, Zn and Cd were 0.462, 0.513, 0.027, 0.0003, 0.027, 0.194, 0.767, 1.558, 1.051 and 2.543 respectively. The sequence of HQ for the elements followed the decreasing order Cd>Mn>Zn>Pb>Cu>Fe>Ni>V=Co>Cr. (Huang et al. 2008) calculate HQ value ( Huang et al.^2008^) for Cd, Ni, Pb, Co, Cr in different vegetables in Khunshan, China and found the sequence of HQ in a decreasing order Pb>Cd>Ni>Co>Cr. The HQ value for all the elements except Mn, Zn and Cd were bellow 1(one), which is considered safe but Mn, Cd, Zn shows a HQ value higher than 1, so there is concern for potential health effect (Huang et al. 2008).

## Conclusions

Metal contamination in Soil is receiving increasing attention all over the world. Principally there are two major pathways for human exposure to soil contamination: soil-plant-human (foodchain pathway) and soil-human (incidental soil ingestion). The present study focused on foodchain pathway. Concentration of different elements in vegetables depends upon the relative level of exposure of plants to the contaminated soil as well as the deposition of toxic elements in the polluted air by sedimentation. In the present study it was found that concentration of Si, Ba, K, Ca, Mg, Fe, Sc, V, Cr, Cu, Zn, As, Mn, Co, Ni, Se, Sr, Mo and Cd in soil samples were higher than the World Average value. As there is no industrial unit near the study area, it seems soil of that area naturally have high concentrations of those elements which may be come from atmospheric deposition by air or other anthropogenic sources.

The elemental concentrations of the studied vegetables varied in different samples and hence variations in elemental concentrations among different varieties reflect the difference in uptake capabilities and their further translocation to the edible portion of the plants. According to (Pendias and Pendias 2000) concentration of the toxic element like As, Co, Cu, Mn, Pb, Se, Ni, V and Zn are below the World Average value (Pendias and Pendias 2000).

Soil-to-plant transfer is one of the key components of human exposure to metals through foodchain. In this study, the soil-to-plant transfer Factor (TF) for various metals and for most common vegetables consumed by human being were calculated (Table 5) and the data showed that the TF values differed significantly between locations and plant species. The difference in TF values between locations may be related to soil nutrient management and soil properties.

The daily intake of metals (Fe, Cu, Mn, Zn, Co, Cr, V, Ni, Pb, Cd) for human with a average body weight 70 kg have been calculated (Table 6) and found that intake of toxic metals from vegetables is not high and within the suggestive values. The calculated Hazard Quotient (HQ) for the above elements except Mn, Zn and Cd is found below 1 which indicates safe with no risk to human health. Higher HQ value for Mn and Zn may not pose risk to human health because these are the essential elements for human. Further in most of the vegetables concentration of Mn is found below the detection level except bean. Zn is an airborne pollutant, so in general it majorly accumulates to open and above-earth vegetables. The daily intake of Cd was estimated 0.178 mg/g and HQ value for Cd calculated was 2.543 which is much higher than the safe value. Cd is very toxic element, its long term exposure to lower level leads to build up in kidneys and possible kidney disease, lung damage and fragile bones. Hypertension, arthritis, diabetes, anaemia, cancer, cardiovascular disease, stroks etc. are its some odd long term results (Arsenic in Drinking Water 1999). Further Cd is not found in all varieties of vegetables. Only Amaranthus, Radish and Cauliflower showed the presence of Cd. It is therefore suggested to consume those vegetables at lower amount in the diet to reduce any toxic effect.

## Authors’ original submitted files for images

Below are the links to the authors’ original submitted files for images. Authors’ original file for figure 1 Authors’ original file for figure 2 Authors’ original file for figure 3
