# Supplementary figures and images for: Transfer of metals from soil to vegetables and possible health risk assessment
Source: Springerplus. 2013 Aug 15;2(1):385. doi: 10.1186/2193-1801-2-385 (PMC3755813; doi:10.1186/2193-1801-2-385)

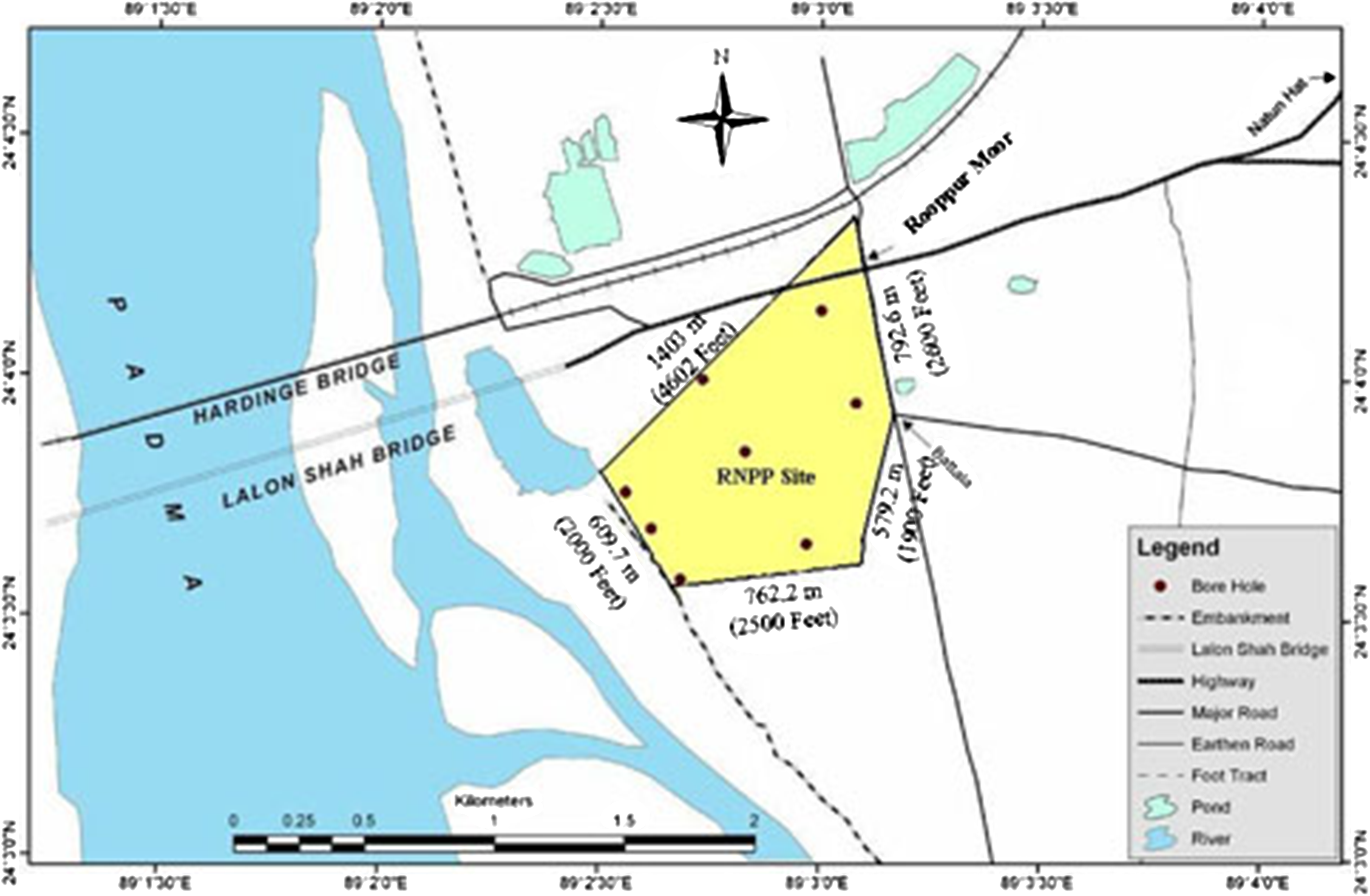

Supplement: Supplementary file 1 — Authors’ original file for figure 1 [file 40064_2013_459_MOESM1_ESM.tiff]

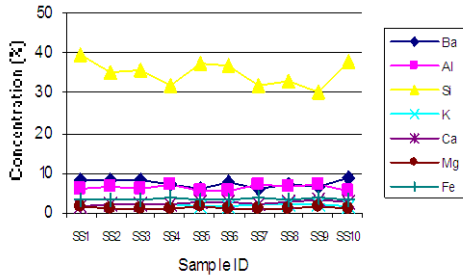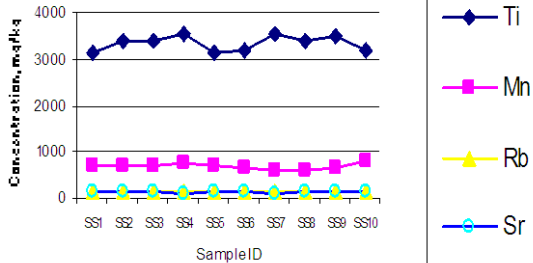

Supplement: Supplementary file 2 — Authors’ original file for figure 2 [file 40064_2013_459_MOESM2_ESM.pdf]

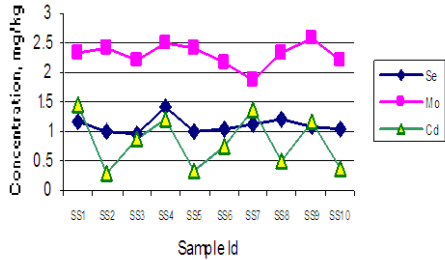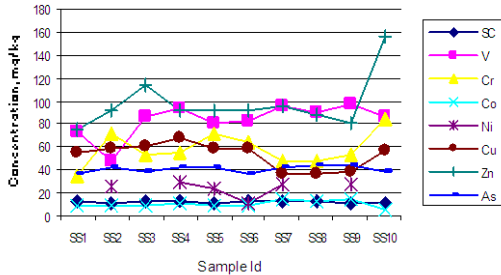

Supplement: Supplementary file 3 — Authors’ original file for figure 3 [file 40064_2013_459_MOESM3_ESM.pdf]
